# Nested-PCR assay for detection of *Schistosoma japonicum* infection in domestic animals

**DOI:** 10.1186/s40249-017-0298-y

**Published:** 2017-04-13

**Authors:** Xin Zhang, Chuan-Chuan He, Jin-Ming Liu, Hao Li, Ke Lu, Zhi-Qiang Fu, Chuan-Gang Zhu, Yi-Ping Liu, Lai-Bao Tong, De-bao Zhou, Li Zha, Yang Hong, Ya-Mei Jin, Jiao-Jiao Lin

**Affiliations:** 1 Shanghai Veterinary Research Institute, Chinese Academy of Agricultural Sciences, Key Laboratory of Animal Parasitology, Ministry of Agriculture, Shanghai, 200241 People’s Republic of China; 2 Anhui Center for Animal Disease Control and Prevention, Hefei, People’s Republic of China; 3 Wangjiang county Center for Animal Husbandry and Veterinary Bureau, Anqing, People’s Republic of China; 4 Dongzhi county Center for Animal Husbandry and Veterinary Bureau, Chizhou, People’s Republic of China; 5 Huangshan Center for Animal Disease Control and Prevention, Huangshan, 341000 People’s Republic of China; 6 Jiangsu Co-innovation Center for Prevention and Control of Important Animal Infectious and Zoonosea, Yangzhou, Jiangsu province 225009 People’s Republic of China

**Keywords:** Schistosomiasis, Domestic animals, Nested-PCR, Diagnosis

## Abstract

**Background:**

Schistosomiasis japonica is a common zoonosis. Domestic animals are the primary source of infection and play an important role in disease transmission. The prevalence and infectivity of this disease in domestic animals in China have significantly decreased and, for this reason, diagnostics with a higher sensitivity have become increasingly necessary. It was reported that polymerase chain reaction (PCR)-based methods could be used to detect schistosome infection in humans and animals and presented a high sensitivity and specificity. The present study aimed to develop a PCR-based method for detection of *Schistosoma japonicum* infection in domestic animals.

**Methods:**

A specific nested-PCR assay was developed to detect *S. japonicum* infection in domestic animals via amplification of a 231-bp DNA fragment of retrotransposon SjR2. The developed assay was first used in sera and dry blood filter paper (DBFP) from goats and buffaloes at different time points of infection. Then, 78 DBFPs from 39 artificially-infected bovines at 14 and 28 days post-infection and 42 DBFPs from schistosome-negative bovines from the city of Huangshan in the Anhui province were used to evaluate the diagnostic validity. Furthermore, this assay was used to detect *S. japonicum* infection in domestic animals in Dongzhi and Wangjiang counties.

**Results:**

The expected PCR product was detected in eggs and adult worms of *S. japonicum* and blood samples from *S. japonicum*-infected goats and water buffaloes, but not from *Fasciola* and *Haemonchus contortus* worms. The nested-PCR assay could detect the target *S. japonicum* DNA in DBFPs from goats and buffaloes after day 3 post-infection. The sensitivity in buffaloes at 14 and 28 days post-infection was 92.30% (36/39) and 100% (39/39), respectively. The specificity was 97.60% (41/42). The positivity rates in Dongzhi and Wangjiang counties were 6.00% and 8.00% in bovines and 22.00% and 16.67% in goats, respectively. The positivity rates in goats in both counties were higher than those in bovines with a significant difference in Dongzhi County but not in Wangjiang County (*P* < 0.05 and *P* = 0.23, respectively).

**Conclusions:**

Our results suggest that the developed nested-PCR assay may be used for the diagnosis of *S. japonicum* infection in domestic animals, and the control of *S. japonicum* infection in goats should be paid more attention.

**Electronic supplementary material:**

The online version of this article (doi:10.1186/s40249-017-0298-y) contains supplementary material, which is available to authorized users.

## Multilingual abstracts

Please see Additional file 1 for translations of the abstract into the six official working languages of the United Nations.

## Background

Schistosomiasis is a public health problem in 74 countries, where approximately 200 million people are symptomatically infected, and 20 million people are heavily infected with different parasite species of the genus *Schistosoma* [1].

Of the five major schistosome species that infect humans, *Schistosoma japonicum* infection in animals is the most significant because it has several reservoir hosts, including humans, buffaloes, cattle, goats, sheep and dogs. Domestic animals, particularly buffaloes, cattle, goats and sheep, are the primary sources of infection and play a vital role in disease transmission. The key strategy for the elimination of schistosomiasis japonica is the eradication of the source of infection [2]. Therefore, the detection of *S. japonicum* in infected domestic animals is critical for the control of this disease.

The miracidium hatching test (MHT) using feces is the most common parasitological test used in China for the identification of *S. japonicum* in domestic animals. Because of the comprehensive control strategy implemented in China from 2004 to block the transmission of *S. japonicum* from cattle/buffaloes and humans to snails [2], the prevalence and intensity of infection of domestic animals with *S. japonicum* decreased to low levels and reached 0.013% in 2014 at the national level [3]. Therefore, the sensitivity and accuracy of MHT are low in this situation because of the low prevalence and infection rates [4, 5]. Antibody detection methods, such as indirect hemagglutination assay (IHA) and rapid strip test, have a high sensitivity but cannot discern active from previous infection [6] and easily cross-react with antibodies from other parasites (parasitic flukes or helminths) [7, 8], because whole crude extracts (e.g., soluble egg antigen (SEA) or soluble worm antigens (SWA) are commonly used as diagnostic antigens. The detection of circulating antigens such as circulating anodic antigens (CAA) in serum or urine seems a promising tool for diagnosis of *Schistosoma* infection in humans [9]. However, most parasitologists have found that the detection of circulating antigens lacks sensitivity in areas of low prevalence [10]. Therefore, the development of highly sensitive diagnostics is essential.

In recent years, various studies have shown that polymerase chain reaction (PCR)-based methods could be used to detect schistosome infection in humans and experimental animals and presented a high sensitivity and specificity [11–13]. Cell-free circulating DNA and some nucleic acid fragments were primarily found in host blood, saliva, semen and urine, and were used as targets for parasite detection [14–16]. Moreover, cell-free circulating DNA has been used as a marker for cancer and prenatal diagnosis [17]. Cell-free circulating DNA of *S. japonicum* is synthesized after host infection. Therefore, DNA extracted from host fluids could be used for early diagnosis [18]. Various PCR-based approaches, including real-time PCR, nested-PCR, and loop-mediated isothermal amplification, have been applied for detection of schistosome infection [14, 19, 20]. However, no previous studies have diagnosed *S. japonicum* infection in domestic animals by using PCR-based methods. In this study, a specific nested-PCR assay was developed to detect *S. japonicum* infection in domestic animals.

## Methods

### Parasite collection

Worms and eggs of *S. japonicum* were collected from the liver and mesenteric vein, respectively, of two adult female New Zealand rabbits artificially infected for 42 days with 1 000 cercariae, as described in previous studies [21, 22]. Worms of *Fasciola* and *Haemonchus contortus* were respectively collected from cattle livers in the Wangjiang County, Anhui Province, and from the goat abomasa in Gong’an County, Hubei Province. All parasites were stored in 70% (v/v) ethanol at room temperature.

### Blood sample collection

The blood samples used in this study included sera and dry blood filter paper (DBFP). The DBFPs were prepared by collecting blood from the ear or jugular vein, depositing each sample on a neutral medium-speed filter paper, letting the sample diffuse in the paper, and drying the DBFP in the shadow. The sera samples were stored at −20 °C, and the DBFPs were stored at room temperature or 4 °C.

Two goats from a non-endemic area of Shanghai were artificially infected with 300 cercariae and treated with an intramuscular injection of 40 mg/kg praziquantel (30% ethanol suspension, based on our previous unpublished studies in mice) on day 138 post-infection. Forty serum samples and 40 DBFPs were simultaneously collected from these animals at 20 different time points from days 3 to 175 post-infection. The detection of *S. japonicum* from days 42 to 175 post-infection in these two goats was done with MHT using 10 g of feces (the collection of blood samples and MHT were executed on the same day) and perfused for worm collection on day 175 post-infection.

A total of 84 DBFPs were collected from 36 schistosome-positive water buffaloes artificially infected with 3 000 cercariae (12 animals) and 1 000 cercariae (24 animals) on days 3 (6 samples from 6 animals), 7 (6 samples from 6 animals), 14 (36 samples), and 28 (36 samples) post-infection. Six DBFPs were collected from three schistosome-positive cattle artificially infected with 600 cercariae on days 14 and 28 post-infection. All these animals were obtained from an area of Nantong city that was not endemic for schistosomiasis.

The standard negative sera and DBFPs used in each test were collected from the animals mentioned above 7 days before infection and confirmed as negative by nested-PCR before the study.

Forty-two DBFPs from schistosome-negative bovine (cattle and buffalo) were collected in July 2016 from Huangshan city in the Anhui province, a former endemic area that eradicated schistosomiasis japonica in 1993.

In July 2016, 180 DBFPs were collected from 100 bovines and 80 goats that grazed freely on marshlands that contained the intermediate host snail, *Oncomelania hupensis*, in the Dongzhi and Wangjiang counties in the Anhui Province. These two counties were endemic for schistosomiasis but achieved the control of transmission (prevalence <1% in bovines and humans) in 2015.

### DNA extraction from parasites and blood samples

Before DNA extraction, approximately 10 *S. japonicum* worms, 5 *H. contortu*s worms, one fragment of a *Fasciola* worm, and 1 to 80 *S. japonicum* eggs were homogenized in 350 μl phosphate buffered saline (0.01 M PBS, pH 7.4). Then, one square centimetre of DBFP from each sample was soaked in 500 μl of PBS for 10 min, and 200 μl of sera were diluted in 150 μl of PBS. Total genomic DNA from parasites and blood samples were extracted from 350 μl of each sample using the AxyPrep™ Multisource Genomic DNA Miniprep Kit (Axygen Scientific, Inc., Union City, CA, USA) according to the manufacturer’s instructions. Each DNA sample was eluted with 100 μl of eluent buffer and stored at −20 °C until further use.

### Nested-PCR

The primers were designed according to the sequence of clone G55A of retrotransposon SjR2 of *S. japonicum* (GenBank Accession No. AF412221) for nested PCR [11]. The outer primers were F2-(5’-GCC TTG CGT CTC TAA TGC T-3’) and R2(5’-GGC GTG TGT CCC TAT CTT-3’), and the inner primers were F1-(5’-TCT AAT GCT AAC GAT TCG AGT-3’) and R1-(5’-TTC CTT ATT TTC ACA AGG TGA-3’) [18]. The expected length of PCR amplicons was 428 bp and 231 bp for the first and second reaction, respectively. The final volume of the first reaction was 25 μl and included 4 μl of DNA template, 12.5 μl of 2× Easy Taq SuperMix (TRANS), 7.9 μl of ddH_2_O, and 200 nM of each primer pair (F2 and R2). The final volume of the second reaction was 25 μl and included 4 μl of DNA template, 12.5 μl of 2× Easy Taq SuperMix (TRANS), 6.5 μl of ddH_2_O, and 200 nM of each primer pair (F1 and R1). The templates of the second reaction were a 10-fold dilution of the amplified products of the first reaction and a 100-fold dilution of the control DNA samples of *S. japonicum* worms. Amplifications in both reactions consisted of an initial denaturing step at 94 °C for 3 min, followed by 35 cycles at 94 °C for 60 s, 60 °C for 60 s, and 72 °C for 60 s with a final extension at 72 °C for 7 min using a PCR system (Eppendorf AG, Hamburg, Germany). The final PCR products were separated by electrophoresis on a 1% agarose gel and stained with DuRed. Some PCR products of the control DNA samples and blood samples were sequenced to confirm that they were the same as subject sequence.

A blank control (PBS), a *S. japonicum* worm DNA control, and a standard negative control were included in each PCR reaction. A nested-PCR result was considered positive in cases in which a 231-bp product was amplified. A sample was considered positive in cases in which the result of the worm DNA control was positive, and the results of the blank and negative controls were negative.

### Statistical analysis

Sensitivity and specificity were assessed by using the following formulas: sensitivity = number of true positives/(No. of true positives + false negatives), and specificity = No. of true negatives/(No. of false positives + true negatives). The differences in the positivity rates between the host species from the DongZhi and Wangjiang counties were analyzed by using chi-square tests in Microsoft Excel 2010, and *P-*values smaller than 0.05 were considered significant.

## Results

### Specificity and sensitivity of the nested-PCR assay

**Fig. 1:**
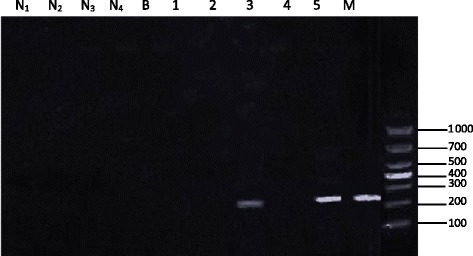
Specificity of nested PCR. N_1_, serum from a non-infected water buffalo; N_2_, DBFP from a non-infected water buffalo; N_3_, serum from a non-infected goat; N_4_, DBFP from a non-infected goat; B, PBS; M, molecular marker; Lanes 1–5: (1) serum from water buffalo assayed on day 3 post-infection; (2) DBFP from water buffalo assayed on day 3 post-infection; (3) serum from goat assayed on day 3 post-infection; (4) DBFP from goat assayed on day 3 post-infection; (5) DNA from an adult *S. japonicum* worm

**Fig. 2:**
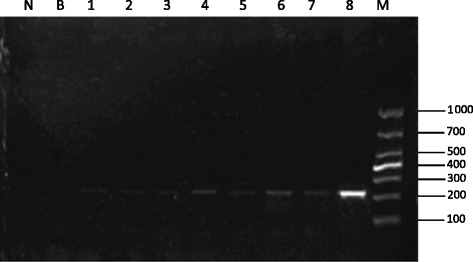
Sensitivity of nested PCR. N, serum of non-infected goats; B, PBS; M, molecular marker; Lanes 1–7: amplification using 1, 2, 5, 10, 20, 40, and 80 *S. japonicum* eggs; (8) DNA from an adult *S. japonicum* worm

The 231-bp DNA fragment was amplified in adult *S. Japonicum* worms (Figs. 1 and 2). However, no PCR products were detected in *Fasciola* and *H. contortus* worms (data not shown). Sensitivity was determined by using a series of 1, 2, 5, 10, 20, 40, and 80 *S. japonicum* eggs in amplification, and the expected product was detected using a single egg (Fig. 2).

### Comparison of nested-PCR results using serum and DBFP

Both sera and DBFP could be successfully used to detect the target *S. japonicum* DNA (Fig. 1). However, the results of sera and DBFPs, which were simultaneously collected from two goats artificially infected for 3 to 175 days, indicated that the rate of detection in DBFPs was higher than in sera (Table 1). *S. japonicum* DNA was detected in all DBFPs but not in sera collected on days 3 and 4 post-infection and days 34 and 37 post-treatment with praziquantel (172 and 175 days post-infection), although some male worms were found in the perfusion on day 175 post-infection.

**Table 1:** Detection of *S. japonicum* DNA in DBFPs and sera from two artificially infected goats and 6 artificially infected buffaloes

Animal	Days post-infection	DBFP		Serum	
		No. of samples^a^	No. of positive samples	No. of samples^a^	No. of positive samples
Goat	3–4	4	4	4	0
	7–22	8	8	8	8
	60–158^b^	20	20	20	20
	163–170^c^	4	4	4	4
	172-175^c^	4	4	4	0
	Total	40	40	40	32
Buffalo	3	6	6	6	0
	7-28	18	18	18	18
	Total	24	24	24	18

^a^ multiple samples collected from two goats and 6 buffaloes at different time points are collectively presented here ;^b^The samples were positive by using MHT; ^c^The samples were collected on days 25 to 37 post-treatment when the MHT results were negative but male worm infection examined by perfusion on final day

### Validity of nested-PCR for diagnosis of bovine schistosomiasis

We detected *S. japonicum* DNA in both DBFP and sera from six artificially infected buffaloes artificially infected with 3 000 cercariae at days 3, 7, 14, and 28 post-infection (Table 1). The results indicated that the expected amplification product was detected in all DBFPs and sera 7 to 28 days post-infection.

The amplification results in 120 bovine DBFPs are shown in Table 2. The sensitivity was 92.30% (36/39) and 100% (39/39) in the samples collected on days 14 and 28 post-infection, respectively, whereas the specificity was 97.60% (41/42).

**Table 2:** Sensitivity and specificity of nested-PCR used for evaluation of *S. japonicum* infection in bovines

Sample	Days post-infection	No. of cases	No. of positive cases	Positivity rate (%)
*S. japonicum*-infected	14	39	36	92.30
	28	39	39	100
Non-infected		42	1	2.4

### Detection of *S. japonicum* infection in domestic animals from endemic regions

The detection results of 180 field DBFP samples collected in endemic areas in Dongzhi and Wangjiang counties in the Anhui province of China are shown in Table 3. The positivity rates in Dongzhi and Wangjiang were 6.00% and 8.00% in bovines and 22.00% and 16.67% in goats, respectively. The positivity rates in goats in both counties were higher than those in bovines, with a significant difference in Dongzhi county but not in Wangjiang county (*P* < 0.05 and *P* = 0.23, respectively).

**Table 3:** Detection of *S. japonicum* DNA in DBFPs collected in endemic areas

Species	County	No. of cases	No. of positive samples	Positivity rate (%)
Bovine	Dongzhi	50	3	6.0
Goat	Dongzhi	50	11	22.0*
Bovine	Wangjiang	50	4	8.0
Goat	Wangjiang	30	5	16.7

*P < 0.05 compared with bovine in Dongzhi

## Discussion

Diagnosis is an important part of the control of schistosomiasis. The current methods used in China for the diagnosis of *S. japonicum* infection in domestic animals are MHT and IHA. However, the diagnostic assays available to date are not ideal because the identification of miracidia in stools has a low sensitivity and antibody detection lacks specificity, which limits the determination of prevalence rates [14]. In this respect, PCR is a potential tool because of its high sensitivity and specificity in the diagnosis of schistosomiasis in humans [23]. *S. japonicum* parasites infect the host through the skin, begin their development in the host, and release DNA fragments into the body fluid of the host, and these fragments can be used as targets for parasite detection [24, 25]. The use of nested-PCR as a diagnostic method has two advantages: first, the templates are amplified twice so that samples can be positive when the template was in limited; second, the reaction is performed using two primer pairs to increase specificity. Herein, we developed a specific nested-PCR assay to detect *S. japonicum* infection in domestic animals.

Our results indicated that both serum and DBFP could be used for diagnosis of *S. japonicum* infection in domestic animals and DBFP was better than serum. The nested-PCR assay in DBFP could amplify the expected product at days 3 and 4 post-infection in both goats and buffaloes and days 34 and 37 after treatment with praziquantel in goats, but not in sera. This result was primarily because of the lower amount of sample template from animals on days 3 and 4 post-infection and days 34 and 37 post-treatment compared with the other days of sample collection and indicates that DBFP may have a higher sensitivity in areas in which the prevalence of infection is lower. On the other hand, the collection in field conditions, transport, and storage of DBFP is easier. We detected *S. japonicum* DNA in DBFPs that were stored at room temperature and 30 °C for 1, 2, 7, 9, 16, and 41 days, and the results indicated that the DBFPs stored at room temperature and 30 °C for 41 days were still positive (Table 4). We also detected the target *S. japonicum* DNA in 19 worm-positive buffalo sera (artificially infected with 1 000 cercariae) that were stored at −80 °C for 1 year and only two samples (10.52%) were positive. This result indicates that the serum DNA stability can be affected by long-term storage and it is best to detect *S. japonicum* infection in domestic animals as early as possible after serum sample collection.

**Table 4:** Detection result of DBFPs with nested-PCR in different storage time and temperature

Storage time (day)	RT (room temperature)		30 °C	
	No. of samples^a^	No. of positive samples	No. of samples^a^	No. of positive samples
1d	2	2	2	2
2d	2	2	2	2
7d	2	2	2	2
9d	2	2	2	2
16d	2	2	2	2
41d	2	2	2	2

^a^ The samples were collected from two goats in different time point

It was reported that parasite DNA is detectable in the serum of rabbits from day 3 post-infection to 3 weeks post-treatment for the monosexual cercariae infection and from day 3 post-infection to 16 weeks post-treatment for the mixed sexual cercariae infection [18]. In our study, the nested-PCR results of goat serum were negative on days 34 and 37 post-treatment. This result indicates that the amount of parasite cell-free DNA in the host circulation may decrease following Praziquantel treatment and the nested-PCR could distinguish between current and past parasitic infections.

Schistosomiasis in domestic animals exhibits a low prevalence and low infection intensity in more epidemic areas with significant progress in schistosomiasis prevention and control, especially in China [2]. In our study, the samples were obtained from artificially infected water buffaloes with 3 000 or 1 000 cercariae, which was far more than that in low-intensity infections. Further studies are needed to accurately detect the animals infected with lower doses of cercariae dose.

The greatest disadvantage of the nested-PCR assay for diagnosis of *S. japonicum* infection in domestic animals was the contamination of laboratory instruments, including PCR tubes and tips, with *S. japonicum* DNA. This problem was solved by leaving all tubes and tips under UV light overnight before use. We also assayed 30 antibody-specific negative buffalo sera obtained from other research groups, and the false positive rate was 16.67% (data not shown). Therefore, it is best to establish a reference laboratory for PCR testing in cases in which the developed nested-PCR assay is used for diagnosis of *S. japonicum* infection in domestic animals.

In 2015, Transmission of schistosomiasis has been controlled in Dongzhi and Wangjiang counties, and the infection rate in humans and bovines is lower than 1% (detected by MHT). However, in our study, the positivity rates in Dongzhi and Wangjiang counties were 6.00%, and 8.00% in bovines and 22.00% and 16.67% in goats, respectively. These results may be because of the higher sensitivity of nested PCR compared to MHT, or the misdetection rate of MHT was higher than that of nested PCR.

The national schistosomiasis control program in China considers only cattle and water buffaloes as significant nonhuman contributors to schistosomiasis transmission on the basis of their size, life expectancy, and infection intensity [26]. The positivity rates in goats in Dongzhi and Wangjiang counties were higher than those in bovines with a significant difference in Dongzhi but not in Wangjiang (*P* < 0.05 and *P* = 0.23, respectively). This result suggests that the control of *S. japonicum* infection in goats should be paid more attention.

## Conclusions

The nested PCR assay we developed could be used to detect *S. japonicum* infection in domestic animals. This assay was effective for the early detection of schistosomiasis in domestic animals.

## Additional file

Additional file 1: Multilingual abstracts in the six official working languages of the United Nations. (PDF 504 kb)

## Abbreviations

DBFP: Dry blood filter paper
IHA: Indirect hemagglutination assay
MHT: Miracidium hatching test
PBS: Phosphate buffered saline
PCR: Polymerase chain reaction
SEA: Soluble egg antigen
SWA: Soluble worm antigens
