# Supplementary material for: Nested-PCR assay for detection of Schistosoma japonicum infection in domestic animals
Source: Infect Dis Poverty. 2017 Apr 13;6:86. doi: 10.1186/s40249-017-0298-y (PMC5390378; doi:10.1186/s40249-017-0298-y)
Supplement: Additional file 1: — Multilingual abstracts in the six official working languages of the United Nations. (PDF 504 kb) [file 40249_2017_298_MOESM1_ESM.pdf]

Translation of the abstract into the six official working languages of the United Nations

إختبار تفاعل سلسلة البوليميرات المتداخل للكشف عن العدوي بالبلهارسيا اليابانية في الحيوانات الداجنة

شين تشانغ، هي تشوان تشوان، جين مينغ ليو، لي هاو، كه لو، تشي تشيانغ فو، تشوان قانغ تشو، يي بينغ ليو، لأي-باو تونغ، دي-باو تشو، لي تشا، يانغ هونغ، يا مي جين، جياو جياو لين

الملخص

الخلفية: تعتبر البلهارسيا اليابانية من الأمراض الحيوانية الشائعة القابلة للإنتقال للبشر وتعتبر الحيوانات الداجنة هي المصدر الرئيسي للعدوى وتلعب دوراً هاما في انتقال المرض للإنسان وتشير الإحصاءات الي أن انتشار العدوى بهذا المرض في الحيوانات الداجنة في الصين قد انخفض إلى حد كبير ولهذا فإن إستخدام أدوات تشخيص عالية الحساسية بات امرا ملحا، وفي هذا السياق أفادت تقارير سابقة أن تفاعل سلسلة البوليميرات يمكن إستخدامه للكشف عن الإصابة بالبلهارسيا في البشر والحيوانات لتميزه بحساسية ودقة عالية لهذا فقد هدفت الدراسة الحالية لتطوير طريقة مبنية على تفاعل سلسلة البوليميرات والتي يمكن إستخدامها في الكشف عن مرض البلهارسيا اليابانية في الحيوانات الداجنة.

الأساليب: تم تطوير طريقة محددة من تفاعل سلسلة البوليميرات المتداخل للكشف عن عدوي البلهارسيا اليابانية في الحيوانات الداجنة من خلال تضخيم قطعة من الحمض النووي طولها 231 زوج من القواعد النيتروجينية من SjR2 وتم إستخدام هذه الطريقة أولا مع ورقة مرشحة من مصل الدم والدم الجاف من الماعز والجاموس عند نقاط زمنية مختلفة من العدوى وبعد ذلك تم إستخدام عدد 78 ورقة ترشيح من 39 من الحيوانات التي تم عدوها بعد 14 يوما و28 يوما من العدوي وتم إستخدام 42 ورقة ترشيح آخري من حيوانات خالية من العدوي من مدينة هوانغشان في مقاطعة آنهوي لتقييم مدي صحة التشخيص وعلاوة على ذلك، استخدمت هذا الطريقة للكشف عن البلهارسيا اليابانية في الحيوانات المحلية في مقاطعات دونجزي ووانجشيانج.

النتائج: تم الكشف عن ناتج تفاعل عديد البلمرة في المتوقع في بيض وديدان البلهارسيا اليابانية وعينات الدم التي تم الحصول عليها من الماعز المصابة بالبلهارسيا وحيوانات جاموس الماء وليس من ديدان الفاشيولا والهايمونتشوس كونتروتوس. إستطاع تفاعل سلسلة البوليميرات المتداخل الكشف عن الحمض النووي للبلهارسيا اليابانية في ورق الترشيح من الماعز والجاموس بعد اليوم الثالث من الإصابة وكانت حساسية الكشف في الجاموس بعد 14 يوم و28 يوما من العدوي 92.30٪ (39/36) و 100٪ (39/39) علي التتابع وكانت التخصصية 97.60٪ (42/41) وكانت المعدلات الإيجابية في مقاطعات دونجزهي ووانججيانج 6.00٪ و 8.00٪ في الأبقار و 22.00٪و 16.67 في الماعز علي التتابع بينما كانت المعدلات الإيجابية في الماعز في كلا المقاطعتان أعلي منها في البقريات مع وجود فارق كبير في مقاطعة دونجزي عن مقاطعة وانجشيانج (,P<0.05 and P=0.23علي التتابع) .

الخلاصة: تشير النتائج الحالية إلى أنه يمكن استخدام تفاعل سلسلة البوليميرات المتداخل الذي تم تطوير لتشخيص العدوى بالبلهارسيا في الحيوانات الداجنة وأن السيطرة على العدوى في الماعز تحتاج الي المزيد من الاهتمام.

Translated from English version into Arabic by Mohamed R. Habib

家畜日本血吸虫病巢式 PCR 检测方法的建立

张欣，何川川，刘金明，李浩，陆珂，傅志强，朱传刚，刘一平，童来宝，周德保，查立，洪炀，金亚美，林矫矫

摘要

引言：日本血吸虫病是一种人兽共患病。家畜是该病的主要传播源，在疾病传播上具有重要意义。目前国内家畜血吸虫病的流行程度及感染强度都已大幅下降，研制更敏感的家畜血吸虫病诊断技术显得越来越重要。据报道，基于 PCR 的各种诊断技术可以用于诊断人和实验动物的日本血吸虫病并且具有较高的敏感性和特异性。本项研究的目的是建立一种诊断家畜日本血吸虫病的 PCR 技术。

方法：通过建立扩增反转录转座子 SJR2 的 231 bp 基因片段的巢式 PCR 技术来诊断家畜日本血吸虫病。建立的巢式 PCR 技术首先用于检测人工感染后不同时间点山羊和水牛的血清及血纸；之后，对来源于 39 头牛人工感染后 14 d 和 28 d 的 78 份血纸、从安徽省黄山市收集的 42 份阴性牛血纸分别进行检测以评估该技术的诊断效果；最后，我们应用该方法对东至和望江的家畜血吸虫病进行了调查。

结果：目的片段可以从日本血吸虫成虫、虫卵以及日本血吸虫感染的山羊和水牛的血液样品中检测，但不能从肝片吸虫和捻转血矛线虫检出。应用血纸进行检测，山羊和水牛在感染第 3 天即可获得阳性结果。对感染后 14 d 和 28 d 的牛血纸进行检测，敏感性分别为 92.30%（36/39）、100%（39/39）。该技术的特异性 97.60%（41/42）。对东至和望江两县的血纸样品进行检测，牛的阳性率分别为 6.00%和 8.00%，山羊的阳性率分别为 22%和 16.67%。两县山羊的阳性率均高于牛的阳性率，东至县具有显著差异但望江县无显著差异（$P < 0.05$ 和 $P = 0.23$）。
结论：本研究结果表明巢式 PCR 可以用于诊断家畜中日本血吸虫病和山羊日本血吸虫病的防控应引起足够重视。

Translated from English version into Chinese by Xin Zhang

Test de PCR emboîtée pour la détection de l’infection à *Schistosoma japonicum* chez les animaux domestiques

Xin Zhang, Chuan-Chuan He, Jin-Ming Liu, Hao Li, Ke Lu, Zhi-Qiang Fu, Chuan-Gang Zhu, Yi-Ping Liu, Lai-Bao Tong, De-bao Zhou, Li Zha, Yang Hong, Ya-Mei Jin, Jiao-Jiao Lin

Résumé
Contexte: La schistosomiase japonica est une zoonose courante. Les animaux domestiques sont la principale source d'infection et jouent un rôle important dans la transmission de la maladie. La prévalence et l'infectiosité de cette maladie chez les animaux domestiques ont considérablement diminué en Chine et, pour cette raison, les tests diagnostiques avec une sensibilité plus élevée sont devenus de plus en plus nécessaires. Il a été rapporté que des méthodes basées sur la polymerase chain réaction (PCR) pouvaient être utilisées pour détecter l'infection par les schistosomes chez l'homme et les animaux et qu’elles présentaient une sensibilité et une spécificité élevées. La présente étude vise à développer une méthode basée sur la PCR pour la détection de l'infection par *Schistosoma japonicum* chez les animaux domestiques.
Méthodes: Un test spécifique de PCR emboîtée a été développé pour détecter l'infection par *S. japonicum* chez des animaux domestiques via l'amplification d'un fragment d'ADN de 231 pb du rétrotransposon SjR2. Le test développé a d'abord été utilisé sur des sérums et du sang sec sur papier buvard (PB) de chèvres et de buffles à différents moments de l'infection. Ensuite, nous avons utilisé 78 PB de 39 bovins artificiellement infectés à 14 et 28 jours après l’infection et 42 PB de bovins négatifs a l’infection par schistosome de la ville de Huangshan dans la province d'Anhui pour évaluer la validité diagnostique. En outre, ce test a été utilisé pour dépister l'infection par *S. japonicum* chez les animaux domestiques dans les comtés de Dongzhi et Wangjiang.
Résultats: Le produit de PCR attendu a été détecté dans les œufs, les vers adultes de *S. japonicum* et dans des échantillons de sang provenant de chèvres et de buffles d’eau infectées par *S. japonicum*, mais pas dans les vers de *Fasciola* et *Haemonchus contortus*. Le test de PCR emboîtée pouvait détecter l'ADN ciblé de *S. japonicum* dans des PB provenant de chèvres et de buffles après le 3ème jour suivant l'infection. La sensibilité chez les buffles 14 et 28 jours après l'infection était respectivement de 92,30 % (36/39) et de 100 % (39/39). La spécificité était de 97,60 % (41/42). Les taux de positivité dans les comtés de Dongzhi et Wangjiang étaient respectivement de 6,0 % et 8,0 % chez les bovins et de 22,0 % et 16,67 % chez les caprins. Les taux de positivité chez les chèvres dans les deux comtés étaient plus élevés que chez les bovins avec une différence significative dans le comté de Dongzhi mais pas dans le comté de Wangjiang (P <0,05 et P = 0,23, respectivement).
Conclusions: Nos résultats suggèrent que le test de PCR emboîtée développé peut être utilisé pour le diagnostic de l'infection de *S. japonicum* chez les animaux domestiques, et que le contrôle de l'infection par *S. japonicum* chez les chèvres devrait être mieux prise en compte.

Translated from English version into French by GARBA DJIRMAY Amadou

Анализ методом гнездовой ПЦР для обнаружения инфекции вызываемой *Schistosoma japonicum* у домашних животных

Xin Zhang, Chuan-Chuan He, Jin-Ming Liu, Hao Li, Ke Lu, Zhi-Qiang Fu, Chuan-Gang Zhu, Yi-Ping Liu, Lai-Bao Tong, De-bao Zhou, Li Zha, Yang Hong, Ya-Mei Jin, Jiao-Jiao Lin

Аннотация

Справочная информация: Японский шистосомоз является распространённым зоонозом. Домашние животные являются основным источником инфицирования и играют важную роль в передаче болезни. Распространенность и инфективность этого заболевания у домашних животных в Китае значительно снизились, и по этой причине диагностика с более высокой чувствительностью становится все более необходимой. Сообщалось, что анализ методом полимеразной цепной реакции (ПЦР) может быть использован для обнаружения заражения шистосомами человека и животных и что данный метод показывает высокую чувствительность и специфичность. Настоящее исследование имеет целью разработку метода на основе ПЦР для обнаружения инфекции *Schistosoma japonicum* у домашних животных.

Методология: Был разработан специфический анализ методом гнездовой ПЦР с целью обнаружения инфекции *S. japonicum* у домашних животных путем амплификации во фрагментах ДНК длиной 231 пар оснований ретротранспозонов SjR2. Разработанный анализ был впервые применен на козах и буйволах на разных стадиях заражения с использованием сыворотки крови и фильтровальной бумаги для метода сухой капли крови (DBFP). Затем, 78 образцов по методу DBFP от 39 искусственно зараженных быков на 14 и 28 день после инфицирования и 42 образцов DBFPs от быков с отрицательным показателем на наличие шистосом из города Хуаншань в провинции Аньхой были использованы для определения диагностической валидности. Кроме того, этот анализ был использован для выявления инфекции *S. japonicum* у домашних животных в уездах Дунчжи и Ванцзян.

Итоги: Ожидаемые продукты ПЦР были обнаружены в яйцах и половозрелых червях *S. japonicum*, а также в образцах крови коз и буйволов, инфицированных *S. japonicum*, но не гельминтами Fasciola и Haemonchus contortus. Анализ методом гнездовой ПЦР может обнаружить искомый ДНК *S. japonicum* в пробах DBFP коз и буйволов по истечению 3-х дней после инфицирования. Чувствительность в случае буйволов на 14 и 28 день после заражения составила 92.30% (36/39) и 100% (39/39), соответственно. Специфичность составила 97.60% (41/42). Количество положительных анализов в уездах Дунчжи и Ванцзян составило 6,00% и 8,00% у быков и 22.00% и 16,67% у коз, соответственно. Доля положительных анализов у коз в обоих уездах была выше, чем у быков, с существенной разницей в уезде Дунчжи, но не в уезде Ванцзян ($P < 0.05$ и $P = 0.23$, соответственно).

Выводы: Наши результаты показывают, что разработанный анализ методом гнездовой ПЦР может быть использован для диагностики инфецирования *S. japonicum* домашних животных и что следует уделять больше внимание контролю над инфекцией *S. japonicum* у коз.

Translated from English version into Russian by Dmitry Esin

Prueba de PCR anidada para la detección de infección por *Schistosoma japonicum* en animales domésticos

Xin Zhang, Chuan-Chuan He, Jin-Ming Liu, Hao Li, Ke Lu, Zhi-Qiang Fu, Chuan-Gang Zhu, Yi-Ping Liu, Lai-Bao Tong, De-bao Zhou, Li Zha, Yang Hong, Ya-Mei Jin, Jiao-Jiao Lin

RESUMEN

Antecedentes: La schistosomiasis japónica es una zoonosis común. Los animales domésticos son la principal fuente de infección y juegan un papel importante en la transmisión de la enfermedad. La prevalencia e infectividad de esta enfermedad en animales domésticos en China han disminuido significativamente y, por esta razón, se hacen cada vez más necesarios los diagnósticos con una mayor sensibilidad. Se reportó que los métodos basados en la reacción en cadena de la polimerasa (PCR) podrían ser utilizados para detectar la infección por schistosomas en humanos y animales, presentando una alta sensibilidad y especificidad. El presente estudio tuvo como objetivo desarrollar un método basado en la PCR para la detección de la infección por *Schistosoma japonicum* en animales domésticos.
Métodos: Se desarrolló un ensayo específico de PCR anidada para detectar la infección por S. japonicum en animales domésticos mediante la amplificación de un fragmento de ADN de 231 pb del retrotransposón SjR2. El ensayo desarrollado se utilizó por primera vez en sueros y en sangre seca en papel de filtro (DBFP) de cabras y búfalos en diferentes momentos de la infección. A continuación, se utilizaron 78 DBFP de 39 bovinos infectados artificialmente a los 14 y 28 días después de la infección y 42 DBFP de bovinos schsitosoma-negativos procedentes de la ciudad de Huangshan, en la provincia de Anhui, para evaluar la validez del diagnóstico. Además, esta prueba se utilizó para detectar la infección por S. japonicum en animales domésticos en los condados de Dongzhi y Wangjiang.
Resultados: Se detectó el producto de PCR esperado en huevos y vermes adultos de *S. japonicum* y en muestras de sangre de cabras y búfalos infectados con *S. japonicum*, pero no de vermes de *Fasciola* y *Haemonchus contortus*. La prueba de PCR anidada podría detectar el ADN diana de *S. japonicum* en DBFPs de cabras y búfalos después del tercer día post-infección. La sensibilidad en búfalos a los 14 y 28 días post-infección fue del 92,30% (36/39) y del 100% (39/39), respectivamente. La especificidad fue del 97,60% (41/42). Las tasas de positividad en los condados de Dongzhi y Wangjiang fueron del 6,00% y 8,00% en bovinos y del 22,00% y 16,67% en cabras, respectivamente. Las tasas de positividad en cabras en ambos condados fueron mayores que en bovinos con una diferencia significativa en el condado de Dongzhi pero no en el condado de Wangjiang ($P < 0,05$ y $P = 0,23$, respectivamente).
Conclusiones: Nuestros resultados sugieren que la prueba de PCR anillada desarrollada puede ser utilizada para el diagnóstico de la infección por *S. japonicum* en animales domésticos y que debe prestarse más atención al control de la infección por *S. japonicum* en cabras.

Translated from English version into Spanish by Màrius V. Fuentes
